# Corrections to “Thermodynamic and Chemical Kinetic Parameters in Ammonia Oxidation: A Comparison of Recent Studies and Parameter Recommendations”

**DOI:** 10.1021/acs.energyfuels.5c00538

**Published:** 2025-03-04

**Authors:** Alon Grinberg Dana, Kfir Kaplan, Michal Keslin, Chuangchuang Cao, William H. Green

The following corrections address inaccuracies in our original paper. While these revisions refine specific details, they do not affect the overall conclusions of our study. We sincerely appreciate Prof. Alexander Konnov for his careful reading and for highlighting some of these issues.

## Correction 1

In Table 3 (p. 22492), there was a typographical error in the value reported for Δ*H*_*f*,298*K*_^*o*^ of HNO_3_. The correct value is –134.1 kJ/mol, taken from ATCT v. 1.202.

## Correction 2

In Tables S3 (p. S18) and S4 (p. S23) of the Supporting Information, the thermodynamic properties of N_2_H_3_ were reported incorrectly. The correct values are as follows:

**For Table S3:**

N2H3 H 3N 2 G 298.000 2500.000 873.91 1

2.54229000E+00 1.10855000E-02-5.70181000E-06 1.52552000E-09-1.67311000E-13 2

2.57459000E+04 1.09370000E+01 2.47040000E+00 1.02434000E-02-2.24608000E-06 3

–2.64426000E-09 1.46424000E-12 2.58032000E+04 1.15301000E+01 4

**For Table S4**:

- name: N2H3(1)

composition: {H: 3, N: 2}

thermo:

model: NASA7

temperature-ranges: [298.0, 873.91, 2500.0]

data:

- [2.4704, 0.0102434, -2.24608e-06, -2.64426e-09, 1.46424e-12, 2.58032e+04, 11.5301]

- [2.54229, 0.0110855, -5.70181e-06, 1.52552e-09, -1.67311e-13, 2.57459e+04, 10.937]

note: ′N[NH]′

## Correction 3

In Tables S5 (p. S31) and S6 (p. S33) of the Supporting Information, the activation energy for the reaction *HNO*_2_⇌*HONO* was given in kJ/mol, and not in kcal/mol like the other rate coefficients in these tables. The corrected values are as follows.

**For Table S5:**

HNO2<=>HONO 1.000e+00 0.000 0.000

PLOG/0.100000 1.090e+47 - 11.480 52.15 /

PLOG/0.215400 1.600e+44 - 10.630 50.79 /

PLOG/0.464100 2.010e+41 - 9.740 49.45 /

PLOG/1.000000 1.650e+38 - 8.790 48.16 /

PLOG/2.154000 7.490e+34 - 7.730 46.87 /

PLOG/4.641000 2.320e+31 - 6.600 45.67 /

PLOG/10.000000 9.050e+27 - 5.470 44.67 /

PLOG/21.540000 8.530e+24 - 4.440 43.88 /

PLOG/46.410000 2.730e+22 - 3.550 43.38 /

PLOG/100.000000 2.670e+20 - 2.800 43.12 /

**For Table S6:**

- equation: HNO2<=>HONO

type: pressure-dependent-Arrhenius

rate-constants:

- {P: 0.1 atm, A: 1.09e+47, b: -11.48, Ea: 52.15}

- {P: 0.2154 atm, A: 1.6e+44, b: -10.63, Ea: 50.79}

- {P: 0.4641 atm, A: 2.01e+41, b: -9.74, Ea: 49.45}

- {P: 1.0 atm, A: 1.65e+38, b: -8.79, Ea: 48.16}

- {P: 2.154 atm, A: 7.49e+34, b: -7.73, Ea: 46.87}

- {P: 4.641 atm, A: 2.32e+31, b: -6.6, Ea: 45.67}

- {P: 10.0 atm, A: 9.05e+27, b: -5.47, Ea: 44.67}

- {P: 21.54 atm, A: 8.53e+24, b: -4.44, Ea: 43.88}

- {P: 46.41 atm, A: 2.73e+22, b: -3.55, Ea: 43.38}

- {P: 100.0 atm, A: 2.67e+20, b: -2.8, Ea: 43.12}

## Correction 4

In Tables S5 (p. S31) and S6 (p. S33) of the Supporting Information, two rate coefficients were taken from a prepublished version (10.26434/chemrxiv-2024-r4fj6) of the work “Pressure-Dependent Kinetic Analysis of the N_2_H_3_ Potential Energy Surface” by Keslin et al. The updated rate coefficients from the final published version (10.1039/D4CP03837A) are provided below:

**For Table S5**:

N2H3(+M)<=>N2H2+H(+M) 1.000e+00 0.000 0.000

TCHEB/300.000 3000.000 /

PCHEB/0.010 98.692 /

CHEB/6 4/

CHEB/ -6.79362 0.813487 -0.140865 0.0133607 /

CHEB/14.8198 0.820276 0.00147819 -0.02546 /

CHEB/ -0.591313 0.239502 0.0625767 -0.0016977 /

CHEB/ -0.304886 0.0320388 0.024277 0.00936021 /

CHEB/ -0.133801 -0.0135305 -2.01909e-06 0.00536465 /

CHEB/ -0.0490817 -0.015718 -0.00813631 0.00114971 /

NH(T)+NH2(+M)<=>N2H3(+M) 1.000e+00 0.000 0.000

TCHEB/300.000 3000.000 /

PCHEB/0.010 98.692 /

CHEB/6 4/

CHEB/1.090e+01 1.961e+00 -2.649e-02 -1.389e-02 /

CHEB/ -1.325e+00 4.625e-02 3.087e-02 1.595e-02 /

CHEB/ -4.545e-01 -4.938e-03 -2.977e-03 -1.239e-03 /

CHEB/ -1.818e-01 -1.975e-03 -1.405e-03 -8.063e-04 /

CHEB/ -6.686e-02 -3.889e-04 -2.866e-04 -1.738e-04 /

CHEB/ -1.792e-02 -9.773e-05 -6.817e-05 -3.804e-05 /

**For Table S6**:

- equation: N2H3<=>N2H2 + H

type: Chebyshev

temperature-range: [300.0, 3000.0]

pressure-range: [0.01 atm, 98.692 atm]

data:

- [-6.79362, 0.813487, -0.140865, 0.0133607]

- [14.8198, 0.820276, 0.00147819, -0.02546]

- [-0.591313, 0.239502, 0.0625767, -0.0016977]

- [-0.304886, 0.0320388, 0.024277, 0.00936021]

- [-0.133801, -0.0135305, -2.01909e-06, 0.00536465]

- [-0.0490817, -0.015718, -0.00813631, 0.00114971]

- equation: NH2 + NH(T)<=>N2H3

type: Chebyshev

temperature-range: [300.0, 3000.0]

pressure-range: [0.01 atm, 98.692 atm]

data:

- [10.8963, 1.96064, -0.0264904, -0.0138879]

- [-1.325, 0.046248, 0.0308719, 0.0159473]

- [-0.45446, -0.00493753, -0.00297691, -0.00123863]

- [-0.181784, -0.00197523, -0.00140519, -0.000806267]

- [-0.0668559, -0.000388914, -0.000286631, -0.000173789]

- [-0.0179225, -9.77336e-05, -6.81701e-05, -3.80433e-05]

